# Antibodies in the Diagnosis, Prognosis, and Prediction of Psychotic Disorders

**DOI:** 10.1093/schbul/sby021

**Published:** 2018-02-21

**Authors:** Thomas A Pollak, Jonathan P Rogers, Robert G Nagele, Mark Peakman, James M Stone, Anthony S David, Philip McGuire

**Affiliations:** 1 Department of Psychosis Studies, Institute of Psychiatry, Psychology and Neuroscience, King’s College London, London, UK; 2 Biomarker Discovery Center, New Jersey Institute for Successful Aging, Rowan University School of Osteopathic Medicine, Stratford, NJ; 3 Department of Immunobiology, Faculty of Life Sciences & Medicine, King’s College London, London, UK; 4 Department of Neuroimaging, Centre for Neuroimaging Sciences, Institute of Psychiatry, Psychology and Neuroscience, King’s College London, London, UK; 5 Joint first authors

**Keywords:** psychosis, schizophrenia, antibody, biomarker, inflammation

## Abstract

Blood-based biomarker discovery for psychotic disorders has yet to impact upon routine clinical practice. In physical disorders antibodies have established roles as diagnostic, prognostic and predictive (theranostic) biomarkers, particularly in disorders thought to have a substantial autoimmune or infective aetiology. Two approaches to antibody biomarker identification are distinguished: a “top-down” approach, in which antibodies to specific antigens are sought based on the known function of the antigen and its putative role in the disorder, and emerging “bottom-up” or “omics” approaches that are agnostic as to the significance of any one antigen, using high-throughput arrays to identify distinctive components of the antibody repertoire. Here we review the evidence for antibodies (to self-antigens as well as infectious organism and dietary antigens) as biomarkers of diagnosis, prognosis, and treatment response in psychotic disorders. Neuronal autoantibodies have current, and increasing, clinical utility in the diagnosis of organic or atypical psychosis syndromes. Antibodies to selected infectious agents show some promise in predicting cognitive impairment and possibly other symptom domains (eg, suicidality) within psychotic disorders. Finally, infectious antibodies and neuronal and other autoantibodies have recently emerged as potential biomarkers of response to anti-infective therapies, immunotherapies, or other novel therapeutic strategies in psychotic disorders, and have a clear role in stratifying patients for future clinical trials. As in nonpsychiatric disorders, combining biomarkers and large-scale use of “bottom-up” approaches to biomarker identification are likely to maximize the eventual clinical utility of antibody biomarkers in psychotic disorders.

## Introduction

According to the World Health Organization, psychotic disorders are the eighth most significant cause of global disability-adjusted life years among 15- to 44-year-olds, ranking above violence, hearing loss, and war.^1^ As well as the substantial loss of quality of life in schizophrenia, it is increasingly recognized that individuals with psychotic disorders have a reduced life expectancy, with one systematic review noting a standardized mortality ratio of 2.58.^2^ These disorders also place an enormous burden on relatives and carers.^3^

Clinical medicine is entering an era in which there is an ever-greater emphasis on the early identification and prevention of disease^4^ and the development of personalized treatment approaches.^5^ As such, biomarkers, which can be diagnostic, prognostic, or predictive, are currently being researched extensively across every area of medicine and psychotic disorders like schizophrenia are no exception. A fundamental problem in the management of psychosis is that outcomes are not predictable on clinical grounds. For example, it is not possible to predict which individuals with prodromal symptoms will develop psychosis, or whether a patient with psychosis will respond to conventional treatment.^6–8^ Hitherto, approaches toward identifying biomarkers have included postmortem studies, structural and functional neuroimaging, proteomics, transcriptomics, metabolomics, and epigenetics. However, these methods have been plagued by small effect sizes, population heterogeneity, and a consequent need for very large samples.^9^

Immune biomarkers are now used widely in relation to physical disease, both systemic and CNS-restricted, to improve clinical understanding and management, reflecting an increasing awareness of the involvement of the immune response in aspects of many diseases. With the recognition that psychotic disorders, too, have considerable immune involvement, it is likely that immune biomarkers will play a role in the biological psychiatry of the future. Here we review the evidence for antibodies as diagnostic, prognostic, and predictive biomarkers in psychotic disorders (table 1).

**Table 1.** Antibodies with Diagnostic, Prognostic, and Predictive Potential in Psychotic Disorders)

Name	Detection method	Diagnostic	Prognostic	Predictive	Comments
		Diagnostic antibodies assist in the identification of a form of organic psychosis	Prognostic antibodies are associated with a particular disorder trajectory	Predictive (or theranostic) antibodies are associated with a response to a particular treatment	
ANA	ELISA	A nonspecific, highly sensitive test for SLE.^37^ SLE can have neurological involvement that presents as psychosis			ANA may be more common in schizophrenia than in controls^131^ (although see ^132^) Ribosomal P antibodies and antibodies to NMDAR NR2 have been associated with neuropsychiatric SLE, but findings are inconsistent^71^
Anti–double-stranded DNA (anti-dsDNA)	ELISA	A specific but insensitive test for SLE^37^			
NR1 subunit of NMDAR	CBA	Associated with NMDAR encephalitis, a distinct form of autoimmune encephalitis that presents with psychosis^66^		Case series and reports suggest patients with acute psychosis and these antibodies may respond to immunotherapy including high-dose steroids, plasma exchange, IVIG, and rituximab^133^ Patients with these antibodies may have an adverse response to antipsychotics^105,134^	NMDAR encephalitis can present with isolated psychiatric symptoms^135^ Seropositivity is insufficient for the diagnosis of NMDAR encephalitis; requires a CSF antibody or confirmatory serum immunoassays^69^ Serum antibodies present in 3%–10% of established schizophrenia and in first- episode psychosis^26^; note some assays also detect in healthy controls
HSV-1			Associated with cognitive deficits in psychosis^80–82,136^ Associated with death from natural causes in schizophrenia^86^	In HSV-1-positive patients with schizophrenia, valaciclovir improved symptoms^114^	Associations between maternal exposure and schizophrenia^20^
CMV			Associated with cognitive deficits in psychosis^84,137^ Associated with suicide attempts in schizophrenia^88^	Improvement in seropositive patients when treated with valaciclovir,^112^ but not replicated in an RCT^113^	
Toxoplasma			Associated with cognitive deficits in psychosis^81,85^ Associated with death from natural causes in schizophrenia^87^ Associated with suicide attempts in schizophrenia^90^	4 RCTs have found that anti- toxoplasma therapy in schizophrenia does not affect psychotic symptoms, but one study found a reduction in negative symptoms and CGI^138^	Associated with schizophrenia^139^
EBV			Associated with death from natural causes in schizophrenia^86^		
TPO and thyroglobulin		Associated with Hashimoto’s encephalopathy,^108^ which can present with psychosis		Case report of schizophrenia- like illness responding well to immunosuppression^110^	Thyroid antibodies detected at high rates in subjects with schizophreniform illnesses^111^
Anti-gliadin and anti-transglutaminase				Case reports and series suggest seropositive patients with schizophrenia have symptomatic benefit from gluten-free diets^122,124,140^	Associated with celiac disease, which shows an epidemiological association with schizophrenia^141^
Folate receptor antibodies		Associated with cerebral folate deficiency		Seropositive patients with schizophrenia improved with folinic acid supplementation^106^	Detected at higher rates than controls in treatment-resistant schizophrenia^106^

*Note*: ANA, Antinuclear Antibody; NMDAR, N-Methyl-d-Aspartate Receptor; HSV-1, Herpes Simplex Virus 1; CMV, Cytomegalovirus; EBV, Epstein-Barr Virus; TPO, thyroid peroxidase; ELISA, Enzyme-Linked Immunosorbent Assay; SLE, Systemic Lupus Erythematosus; RCT, Randomized Controlled Trial.

### Immune and Inflammatory Mechanisms are Implicated in Psychotic Disorders

Autoimmune diseases occur when tissue destruction is mediated by self-antigen directed antibodies or T-cells.^10^ To the extent that we understand the pathogenesis of psychotic disorders, there is insufficient evidence to claim that they are autoimmune in origin.^11^ There is, however, a consensus that psychotic disorders are heterogeneous, in many cases with complex multifactorial aetiologies. Some authors have proposed the categories of “primary” or idiopathic, and secondary psychoses, that is, those in which there is a clearly identifiable “organic” cause (eg, temporal lobe epilepsy, the 22q11 deletion syndrome).^12^ Other authors have gone further still, arguing that the term “schizophrenia” should be replaced with a broader concept such as “psychosis spectrum disorder.”^13^ Nonetheless the evidence for some role for both adaptive and innate immune processes in the aetiology of some of these disorders continues to mount.^14^ Recent genome-wide association studies (GWAS) point toward immune-related loci in schizophrenia with multiple susceptibility genes identified on the major histocompatibility complex.^15^ When GWAS findings are grouped by known molecular pathways, several related to immunity and inflammation are implicated.^16^ Elevated serum levels of some inflammatory cytokines characterize the acute phase of psychotic disorders,^17^ and there is preliminary evidence that cytokines may have a role in predicting illness course or treatment response in individuals in the earliest stages of psychotic disorders.^18,19^

Prefiguring these more recent studies of inflammatory markers in psychotic disorders, associations between infectious organisms and psychotic disorders have been described for over a century. Since the studies that linked an increased incidence of schizophrenia to epidemic influenza infection during the second trimester, associations have been noted between maternal exposure to influenza, *Toxoplasma gondii* and herpes simplex virus (HSV).^20^ In terms of later neurodevelopment, there is evidence that childhood and even adult infection with *T. gondii* and other organisms is associated with psychosis.^21–23^

Epidemiological studies have also borne out the association between psychotic disorders and autoimmune disease. Rates of autoimmune disorders such as celiac disease, Graves’ disease, systemic lupus erythematosus (SLE), multiple sclerosis, autoimmune hepatitis, and psoriasis are higher in those with schizophrenia.^24^ Moreover, a family history of multiple sclerosis, psoriasis, Sjögren’s syndrome, dermatopolymyositis, or autoimmune hepatitis is associated with a greater risk of schizophrenia.^24^ Severe infections and autoimmune diseases show independent associations with schizophrenia, but they also have a synergistic effect on the risk.^25^

Focusing more specifically on humoral immunity, the notion that psychosis might be caused by a pathogenic antibody has a long history. A systematic review demonstrated that among patients with established schizophrenia, 20 autoantibodies (including antinuclear antibody [ANA], anti-cardiolipin, anti-N-methyl-d-aspartate receptor [NMDAR], and anti-serotonin) were present at higher rates than among controls; rates of anticardiolipin and anti-NMDAR antibodies were also present in patients with first-episode psychosis.^26^ Even among the unaffected first-degree relatives of patients with schizophrenia, a higher prevalence of some autoantibodies has been observed.^26^ However, the mere presence of an antibody does not imply pathogenicity.

Initially, associations with psychosis have been described for antibodies that reacted with entire brain regions.^27^ However, the reactivities of such antibodies are nonspecific enough that they are unlikely to be helpful in understanding pathogenesis or in distinguishing psychotic disorders from other disorders. A more refined hypothesis is that patients with psychotic disorders (or a subgroup thereof) have pathogenic antibodies against specific neuronal cell surface proteins such as the NMDAR, and research is ongoing to establish the aetiological and prognostic significance of this.^28–30^

### Antibodies in the Prediction and Stratification of Physical Disease

Antibodies make suitable biomarkers for the prediction of disease because they are relatively easily measured in bodily fluids by a variety of (usually inexpensive) immunoassays. Biomarkers—whether antibodies or otherwise—can have 3 distinct roles in medicine. They may be diagnostic, indicating the presence or absence of a disease (although often their sensitivity and specificity is such that they lend support to or help rule out a disease entity). They may be prognostic, giving information on morbidity, mortality, or another outcome. Finally, they may be predictive, giving information on a patient’s likely response to specific treatments.^31,32^

Where a disorder has an established autoimmune basis, autoantibodies have a clear role as biomarkers, although crucially there is no requirement in any disease for an antibody to be pathogenic (causal) in order for it to have a useful biomarker role. Indeed, many of the biomarkers discussed here are unlikely to be causal. This concept may be the source of some confusion but is of utmost importance. To establish that an antibody is “causal” for a particular disease (ie, necessary and sufficient for the occurrence of disease) requires considerable evidential support in the fulfilment of the so-called Koch-Witebsky postulates, namely: (1) evidence of disease-specific adaptive immune response in the affected target tissue, organ, or blood; (2) passive transfer of antibodies replicates the disease in experimental animals; and (3) elimination of antibodies modifies disease.^10^

If an antibody is not primarily causal, it may still have an associated disease-modifying role and therefore shape phenotype despite not being required for the disease to be present. For example, circulating autoantibodies are the secreted product of a pathway that includes the generation of B lymphocytes bearing autoantibodies as their surface immunoglobulin. These are potent antigen-presenting cells for autoreactive T cells and in this capacity alone are likely to drive immunopathology. Alternatively, antibodies may be raised in response to the primary disease pathology but may not be disease-causing in themselves (ie, “epiphenomenal”) or they may have an even less direct association with the disease pathology (eg, they may associate with a risk factor that in itself is only contributory to the disease). Whether or not an antibody is causal, therefore, is an independent question to that of its utility as a biomarker. If an antibody is clearly causal then this may indicate that it will be a useful biomarker for clinical response to antibody-depleting immunotherapies, but there are many more useful contexts in which an antibody can have a useful biomarker role.

Most autoimmune diseases develop over a long period of time, with a period of subclinical autoreactive tissue damage before the development of overt symptomatology. A paradigmatic example of the development of predictive antibody markers is that of type 1 diabetes (T1D). Based on seminal studies of first-degree relatives who were monitored for the development of T1D over 15 years, the risk of developing the illness is now understood to rise with the number of organ-specific autoantibodies.^33^ Subsequent studies have refined the selection of at-risk individuals by using genetic criteria (HLA typing) and have provided robust risk estimates based on the “burden” of islet cell–specific autoantibodies. For example, children who develop two or more such autoantibodies have a risk of developing T1D in childhood or adolescence of >80%.^34^

In SLE, there are raised levels of various autoantibodies, including ANA, anti-dsDNA, ENA, anti-Ro, anti-La, anti-RNP, and anti-Sm.^35^ ANA is most commonly used as a “screening” test, wherein a positive result will prompt testing of the other disease-associated antibodies. Patients with particular antibodies have an increased risk of developing certain manifestations of SLE.^36^ However, 4%–8% of the healthy population (depending on the threshold used) are positive for ANA and rates are higher in those with multiple comorbidities, so it is not regarded as a specific test.^37,38^ Anti-dsDNA is more specific, but this is at the expense of sensitivity.^37,38^ Similar to T1D, autoantibodies have been shown to predate clinical manifestations in a large proportion of those who subsequently develop the disease with the lag time between seropositivity and diagnosis being as much as 9 years.^39^

Neuromyelitis optica (NMO) is a central nervous system disease characterized by inflammatory optical and spinal lesions. Previously characterized as “optical-spinal” MS, the identification of pathogenic antibodies to the astrocytic water channel aquaporin-4 (AQP4)^40^ heralded a recategorization of the disorder as an independent entity.^41,42^ Specific antibody and B-cell depleting therapies have been shown to be efficacious and AQP4 antibodies have been shown to predate the development of NMO. Furthermore, seropositivity at the time of an initial episode predicts higher relapse rates than seronegative status.^41^ Immunoglobulin access to the CNS is thought to be restricted by the blood-brain barrier (BBB). This suggests that the first step in pathogenesis, in NMO and other in autoantibody-mediated CNS diseases, is BBB disruption: in the case of NMO due either to antibodies directed against AQP4 within the BBB^43^ or to induction of IL-6 production by AQP4-positive astrocytes.^44^ This disruption may allow leakage of AQP4 antibodies into the cerebrospinal fluid (CSF).^45^ We have reviewed the implications of BBB dysregulation in psychosis elsewhere.^46^

Moving beyond antibodies targeting “self” antigens, antibodies to infectious organisms have also been useful as biomarkers in diseases not classically understood to be infectious. For instance, within oncology, Epstein-Barr Virus (EBV) is thought to have a causal role in the development of Burkitt’s lymphoma and Hodgkin’s disease^47^; raised titers of antibodies to EBV are seen in both of these disorders.^48^ Similarly, antibodies to human papilloma virus may have a role in the prediction of outcomes for oropharyngeal squamous cell carcinomas.^49^

As well as being relevant in classically infectious and malignant disease, infectious organism antibodies are also thought to be implicated in autoimmune diseases. In Crohn’s disease, antimicrobial antibody serostatus (including to *Saccharomyces cerevisiae*) before diagnosis predicts subsequent disease course.^50^ The relationship between infection, antibodies to infective pathogens, and autoimmunity is complex: a paradigmatic example is rheumatic fever, a disease that is precipitated by infection by *Streptococcus pyogenes*, a bacterium that usually produces an acute upper respiratory tract infection. In a minority, however, untreated infection with the pathogen results in subsequent weeks in inflammatory disease of the skin, joints, and myocardium. The mechanism thought to underlie this is known as molecular mimicry, whereby pathogens express antigens with similar epitopes to host tissue. This similarity is not sufficient to prevent an adaptive immunological response against the pathogen, but it does mean that once antibodies are formed, they can cross-react with host tissue.^51^ In rheumatic fever, antibodies produced against streptococcal antigens cross-react with cardiac myosin, resulting in myocarditis.^52^ Other examples of autoimmune diseases that may have infective triggers are Graves’ disease (*Yersinia enterocolitica* mimics the TSH receptor) and multiple sclerosis (multiple viruses share epitopes with the myelin basic protein).^51^ Apart from molecular mimicry, there are other proposed mechanisms to explain these associations: for instance, there may exist microbial “superantigens” that could nonspecifically prime T-cells; bacterial endotoxins may cause polyclonal B-cell activation, or self antigen may undergo posttranslational modification to make it more immunogenic.^53^

The examples above largely feature antibodies that were specifically sought either within disease or at-risk cohorts, usually because of previous work demonstrating a plausible mechanistic link between the antigen in question and the disease state. This hypothesis-driven or “top-down” approach to biomarker validation has been successful to a point but, particularly in CNS disorders, has also highlighted an important cautionary lesson: that outside of strictly-defined autoimmune disease in which autoantibodies are thought to be directly pathogenic, “classical” antigenic targets often miss their mark as the most useful biomarkers.^54^ For example, serum autoantibodies to amyloid-β do not show a clear association with Alzheimer’s disease, and a similar lack of clarity characterizes the literature on α-synuclein antibodies in Parkinson’s disease.^54^

**Fig. 1.**
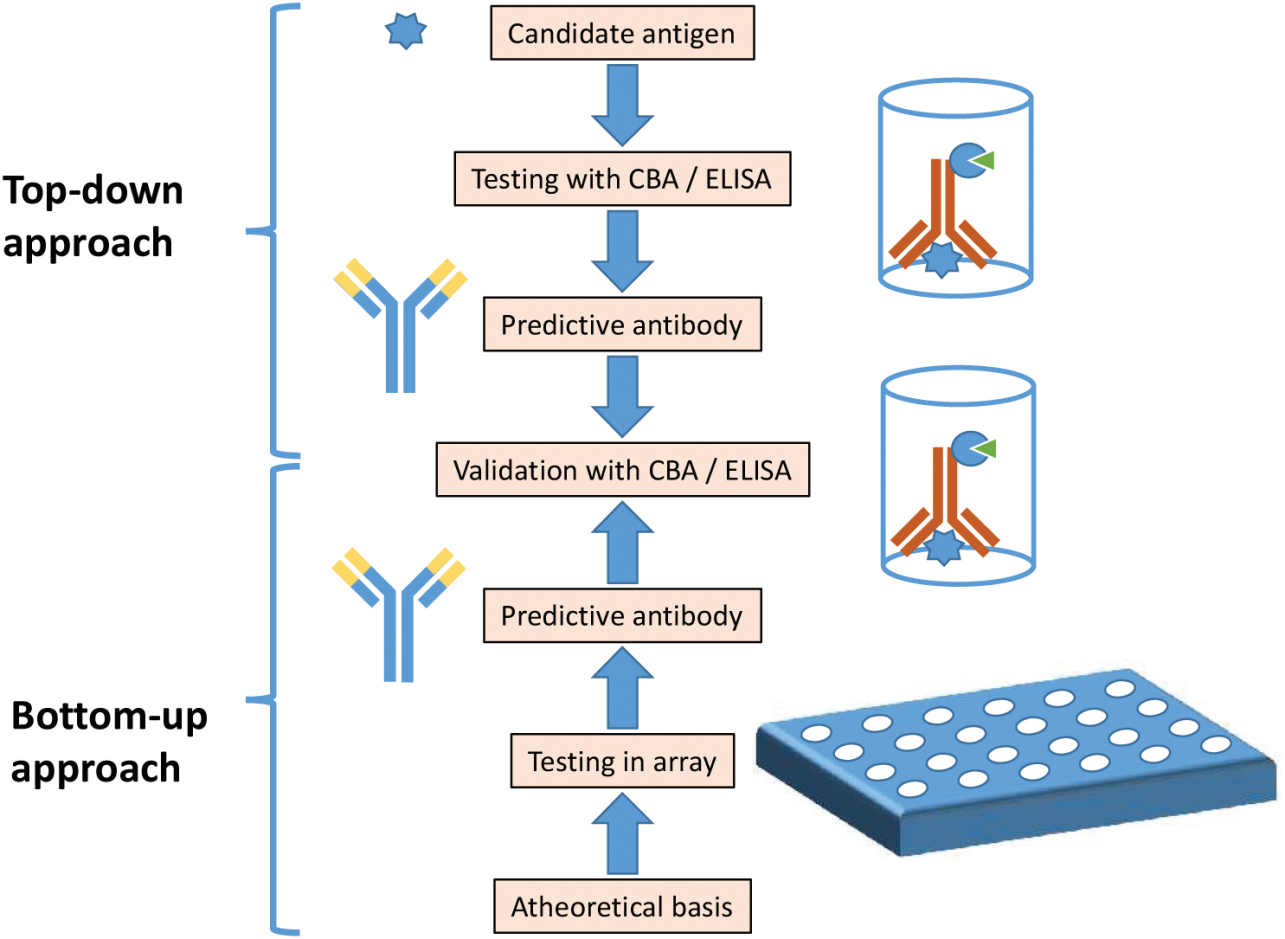
Two approaches to identification of antibodies for psychosis. The top-down approach is hypothesis-driven, based on candidate antigens identified from related disorders, genetic studies or putative neurobiology. The bottom-up approach is hypothesis-neutral and aims to identify predictive antibodies based on a large microarray. Both require validation with a cell-based assay (CBA) or other immunoassays such as enzyme-linked immunosorbent assay (ELISA).

The last decade has seen the emergence of an alternative approach to the development of antibody-based diagnostic and predictive markers. High-throughput immunoassay platforms now allow for the simultaneous testing of antibodies to many thousands of antigenic targets on a single biological sample. This approach remains agnostic as to the potential significance of any one antigen in a given disease. It has been demonstrated that every individual harbors many thousands of autoantibodies directed against self antigens—so-called “natural autoantibodies”—and that the vast majority are not disease-causing. Indeed natural autoantibodies exist in multiple isotypes and with varying affinities in all individuals regardless of age, gender, or disease state, and the production of these autoantibodies is likely to represent a physiological “debris-clearing” response to tissue destruction or damage.^55^ It follows that the autoantibody profile of any individual might reflect any pathological process that is ongoing in that individual and can thus serve as a “readout” of the disease state in question (figure 1).

This approach is starting to be used with some success in neurodegenerative disorders.^54^ For example, using a panel of 50 autoantibodies, researchers were able to accurately distinguish patients with mild cognitive impairment from controls and from those with mild-moderate Alzheimer’s disease.^56^ Another study identified an autoantibody biomarker panel able to distinguish early-stage Parkinson’s disease from disease and non-disease controls and showed promise for staging the disorder.^57^ A similar approach has shown some success in subtyping multiple sclerosis.^58^ These studies provide hope for early diagnosis of neurodegenerative diseases using blood-based biomarkers without the need for expensive and invasive testing and with application in the primary care setting.

As was the case in genetics, where a genome-wide approach has supplanted a “top-down” single-gene approach, it is likely that a “bottom-up” approach to biomarker identification will prove particularly useful in complex disorders, with multifactorial aetiology, where pathology occurs in multiple systems. This is a model with increasing relevance to psychiatry, where simplistic “single receptor” or “single neurotransmitter” models of disease are in decline.

### Special Considerations in Psychosis

#### Diagnostic Antibodies.

Neuroimaging and blood-based biomarkers have thus far failed to completely distinguish psychotic from other psychiatric disorders.^59–61^ Given that there are no adequately sensitive and specific biomarkers for the diagnosis of primary or idiopathic psychotic disorders like schizophrenia, how could antibodies have any diagnostic role in the clinical assessment of patients presenting with psychosis?

One very clear use is in the diagnosis of a so-called organic (or “secondary”) psychotic disorders, which estimates suggest may account for between 3% and 6% of all cases of psychosis.^62,63^ In the last decade, an increasing awareness of autoimmune encephalitis as a differential diagnosis of acute psychosis has led to many clinicians testing for neuronal autoantibodies as part of the initial assessment of patients presenting with a first episode of psychosis or even an acute relapse. The rationale is that early testing might point clinicians toward identifying an immunotherapy-responsive encephalopathy *before* neurological symptoms develop.^64,65^ It is notable in this regard that in the initial series of NMDAR encephalitis, nearly 80% of patients presented initially to mental health services.^66^ Further, it is now understood that autoimmune encephalitides can also present *monosymptomatically*, with only psychiatric symptoms but not neurological symptoms.^64,67,68^ In much the same way that an autoantibody test (for aquaporin 4 antibodies) allowed neurologists to delineate and recategorize (as NMO) a small subgroup of patients with demyelinating disease that were previously subsumed under the broad category of MS,^42^ testing for NMDAR encephalitis may therefore allow psychiatrists to recategorize a proportion of cases that hitherto were subsumed under the category of “schizophrenia.”

In these cases, according to recent diagnostic consensus criteria, antibodies must be present in CSF or there must be other paraclinical evidence suggestive of encephalitis, such as electroencephalography (EEG), neuroimaging, or inflammatory CSF abnormalities.^69^ Therefore, antibody testing cannot *in itself* lead to a diagnostic recategorization in a patient who presents with psychosis without additional symptoms, but it can guide further diagnostic investigation.

This approach does, therefore, leave a potentially large group of patients facing a lack of diagnostic clarity, that is, patients who present with psychotic symptoms and who have a positive serum neuronal autoantibody test result but who do not have EEG, neuroimaging, or CSF abnormalities. These patients have been designated “synaptic and neuronal autoantibody-associated psychiatric syndromes” (SNAps) by Al-Diwani et al^70^ and will be considered in section 3.3.

Other diagnoses of organic psychosis that can be aided by a positive antibody test are most often made in the context of a systemic or CNS disease that can present with psychosis. Examples include SLE, the antiphospholipid syndrome, vasculitis, or neurosyphilis. In these cases, the diagnostic antibody is not usually specific to a psychotic presentation but to the disease more generally. One possible exception is psychosis associated with SLE (or “lupus psychosis”), which may be specifically associated with antibodies to the NR2 subunit of the NMDAR (and shown to cross-react with dsDNA) or with ribosomal P antibodies.^71^

Perhaps surprisingly, antibody-based diagnostics in psychiatry have probably impacted clinical practice most within the pediatric sphere. Pediatric acute-onset neuropsychiatric syndrome (PANS) is a (somewhat contentious) clinical diagnosis defined by the sudden onset of obsessive-compulsive symptoms or eating restrictions in combination with a number of other possible comorbid neuropsychiatric symptoms, and is strongly associated with infection with group A streptococcus and other pathogens. Particularly relevant to this review, about a quarter of patients also present with psychotic symptoms, although these do not form part of the diagnostic criteria.^72^ PANS, along with a related syndrome, pediatric autoimmune neuropsychiatric disorder associated with streptococcal infections (PANDAS), have been variously associated with (putatively causal) antibodies targeting the basal ganglia, the dopamine D1 and D2 receptors, enolase, beta-tubulin, and lysoganglioside-GM1 (lyso-GM1).^73^ The sensitivity and specificity of various antibody-based diagnostics for these disorders is however variable, with a recent study demonstrating that a popular commercially available panel of antibodies (the Cunningham panel) had relatively poor performance in identifying children with clinically defined PANS/PANDAS, with positive results among healthy controls and poor test-retest reliability.^72^

We suggest that a “bottom-up,” single-platform multi-specificity detection approach may show promise in identifying diagnostic biomarkers for psychotic disorders, although to what extent this is possible is of course likely to be dependent on the extent to which the psychotic disorder group is aetiologically heterogenous. By identifying the *panel* of antibodies that most discriminate patients with psychotic disorder from matched healthy or psychiatric controls, it is possible that truly diagnostic biomarker identification can be facilitated.

#### Prognostic Antibodies.

A fundamental challenge in the management of patients with psychosis is that the course of the disorder is remarkably heterogenous. Some patients have a single episode of psychosis and then make a very good recovery, with no further episodes of illness. Others have an episodic course, with successive periods of acute illness and remission. A further subgroup follows a chronic, unremitting course, with a progressive decline in functioning.^74,75^ These different types of patients require very different types and levels of clinical care. At the onset of illness, it is not possible, on the basis of their presenting clinical features, to predict which particular course a patient will follow. There is thus great interest in the potential of biomarkers to help stratify patients with psychosis according to their future clinical course.^6^

Additionally, there is mounting interest in biomarkers of particular domains of impairment in psychotic illnesses; for example, cognitive dysfunction is recognized as an important predictor of outcome in psychosis, and indeed one that is relatively refractory to pharmacotherapy.^76^ As “psychosis” is reconceptualized in the coming decades, it may become apparent that there is a subtype characterized by a progressive course and poor cognitive profile. In this sense, today’s prognostic biomarkers may have a *diagnostic* role in future practice.

Although neuronal autoantibodies, in the context of autoimmune encephalitis, have been linked with poor cognition and functioning over a follow-up period of years,^77,78^ their prognostic role in psychotic disorders has not been assessed.

A significant body of work has associated cognitive deficits in psychosis with antibodies to viruses and other neurotropic pathogens, with the most commonly implicated organisms being HSV-1,^79–82^ cytomegalovirus (CMV),^83,84^ and toxoplasma.^81,85^ Similarly, mortality from natural causes in schizophrenia is associated with antibodies to HSV-1, toxoplasma, and EBV.^86,87^ Finally, suicidality and a history of suicide attempts may be predicted by both the presence and titer of antibodies to CMV and toxoplasma.^88–90^

However, given the relatively high seroprevalences of antibodies to some of these pathogens, and the often small effect sizes in relation to the outcomes of interest, the possibility of confounding is high and it is unlikely that a single positive infective antibody test will have sufficient clinical utility to inform management. A related concern with the use infective antibodies as biomarkers is the extent to which the association with the outcome of interest is due to confounding with ethnicity, socioeconomic status or other potentially relevant environmental factors such as urbanicity, migration status or lifestyle, and behavior.^91–93^

Finally, an unbiased “bottom-up” approach has recently been used for identification of prognostic autoantibodies in first episode psychosis: Zandian et al used microarrays to profile the autoantibody repertoire of first episode psychosis patients and controls. One of the most discriminant autoantibodies, targeting the N-terminal domain of the PAGE (P antigen) protein group, was linked to a 4-fold risk of future diagnosis of schizophrenia. Despite PAGE being a protein that had not previously been associated with psychosis, this pilot study is the first to employ a bottom-up approach for antibody biomarker identification in psychotic disorders, suggesting clear promise for the general approach, and identifying candidate biomarkers that would not have emerged from a hypothesis-driven, top-down approach.^94^

We suggest that a fruitful approach would be the identification of prognostic antibody biomarkers in subjects at clinical risk for psychosis. These individuals have “attenuated” psychotic symptoms, and about a third will progress to frank psychosis within 2 years.^95^ To date, no study has measured neuronal autoantibodies in this group to establish whether they predate the onset of frank psychosis, and whether they confer risk for the subsequent development of a psychotic disorder.

#### Predictive (Theranostic) Antibodies.

A final application for measurement of antibodies in patients with psychosis is to predict response to treatment with antipsychotic medication. In this regard, it is too narrow to consider only the prediction of a positive response (ie, reduction of psychotic symptoms).^8^ Identification of predictors of treatment resistance or of adverse treatment effects is also a priority, as is identification of predictors of response to novel therapies such as immunotherapies. Finally, improvement in symptom domains other than explicitly psychotic symptoms, for example, cognitive deficits, is an important outcome to consider.

Considering the potential predictive role of neuronal autoantibodies, favorable outcomes have been reported when combinations of corticosteroids, plasmapheresis, IVIg, mycophenolate mofetil, or rituximab have been used in a case series in patients with antibodies to the NMDA receptor and no overt neurological signs (ie, patients who do not have autoimmune encephalitis but who would meet Al-Diwani et al’s “SNAps” definition^70^).^96^ Randomized trials of immunotherapy in patients with psychosis and NMDAR and other neuronal autoantibodies are currently ongoing. If positive, and a serum NMDAR antibody test can predict a good immunotherapy-response, the implications for clinical psychiatric practice are potentially transformative.

(NMDAR antibodies have been shown to disrupt neuronal glutamatergic signaling.^97^ It is possible that when detected in individuals with psychosis, then, these individuals may have a primarily glutamatergic rather than dopaminergic pathology. Furthermore, response to antipsychotic treatment in psychosis is associated with dopaminergic pathology and nonresponse with glutamatergic pathology.^98,99^ However, no study to date has assessed glutamatergic and/or dopaminergic function in NMDAR antibody–positive patients with psychosis.)

Interestingly there is some evidence that neuronal autoantibodies may be most frequently identified in subgroups of patients with psychosis that have classically been felt to be more “organic” in nature, for example, childhood-onset psychosis,^100^ postpartum psychosis,^101^ or psychosis associated with epilepsy.^102^ These studies did not specifically assess immunotherapy-response. In the epilepsy literature, there is evidence that the presence of neuronal autoantibodies in chronic refractory “idiopathic” epilepsies indicates an increased likelihood of preferential response to immunotherapies over standard antiepileptic medications,^103^ raising the intriguing parallel possibility that neuronal autoantibody status in these psychoses may index immunotherapy-responsiveness over antipsychotic-responsiveness.

Further, NMDAR antibodies may indicate that a patient will have an adverse response to antipsychotic medications, with increased rates reported of rhabdomyolysis and neuroleptic-malignant syndrome-type reactions as well as extrapyramidal symptoms.^104,105^

Where autoantibodies to specific receptors indicate dysfunction of the associated neurochemical system, these antibodies may represent an opportunity for treatments targeting that system. For example, folate receptor antibodies were described in 15 of 18 patients (83.3%) with refractory schizophrenia, compared with 3.3% of healthy controls. These antibodies were hypothesized to block the receptor and modulate flux of folic acid into and out of the brain in a manner analogous to that seen in infantile-onset cerebral folate deficiency syndrome. Eight seropositive patients were treated with folinic acid supplementation with improvement reported in 7 patients.^106^ No randomized study has yet attempted to replicate this interesting open-label study. In a similar vein, a single case study demonstrating improvement in psychotic symptoms in a woman with chronic schizophrenia, NMDAR antibodies and characteristic EEG abnormalities reported significant improvement with d-serine, an NMDAR co-agonist.^107^

Hashimoto’s encephalopathy (also known as steroid-responsive encephalopathy associated with autoimmune thyroiditis, SREAT) is characterized by diverse neuropsychiatric signs in the presence of thyroid autoantibodies.^108^ Symptoms cannot merely be attributed to thyroid dysfunction.^109^ Endres et al^110^ have recently reported a case of elevated antithyroid peroxidase (TPO) antibody levels in a patient with a schizophrenia-like illness who responded well to immunosuppression with corticosteroids. They also reported antibodies to either TPO or thyroglobulin (TG) in 13 of a series of 100 patients with schizophreniform syndromes,^111^ raising the possibility that TPO and TG antibodies could be used to characterize a group of patients who might respond well to corticosteroid therapy.

Antibodies to infectious antigens may have a special role in personalizing adjunctive treatment for patients with psychotic disorders. An open-label study in 2003 showed an improvement in psychiatric symptoms in patients with schizophrenia who were seropositive for CMV,^112^ but this was not replicated in a randomized double-blind trial.^113^ Prasad et al,^114^ in a placebo-controlled, double-blind randomized controlled trial (RCT) showed that 18 weeks of valacyclovir treatment improved cognition in a number of domains, but not psychotic symptoms, in HSV-1 seropositive patients with schizophrenia.

Despite the evidence of an association between psychotic disorders and antibodies against toxoplasma, these antibodies do not appear to predict symptomatic response to anti-toxoplasma therapy in schizophrenia, insofar as 4 RCTs have failed to find a main effect on psychotic symptoms.^115^ Notably one study did find that artemether treatment was associated with a greater reduction in PANSS negative symptom scores, and in clinical global impression scores, when compared with placebo, in 100 toxoplasma antibody–positive schizophrenia patients, although this was not the primary outcome of the trial.^116^

Recently, dietary antibodies have been implicated in the pathogenesis of some cases of psychotic disorder.^117–120^ Case studies and case series indicate that patients who are seropositive for antibody markers of gluten sensitivity (eg, anti-gliadin, anti-transglutaminase antibodies)^121^ may benefit symptomatically from gluten-free diets^122–124^ although trial evidence is lacking.

### Conclusions and Future Directions

The gold-standard biomarker is one that is highly sensitive, highly specific and noninvasive.^125^ The biomarkers that we have surveyed in relation to psychosis do not currently meet standards that would support their general clinical use as biomarkers for diagnosis or prognostication of typical psychotic disorders, nor for predicting the response of psychotic symptoms to antipsychotic medication. However, there may be a promising role for antibodies in the following situations:

1. diagnosis of organic or atypical psychosis syndromes;
2. clinical course in primary psychotic disorders, particularly as regards cognitive impairment;
3. prediction of likelihood of response to immunotherapy or other novel therapeutic strategies.

Regarding neuronal autoantibodies in particular, considerable further work is required to evaluate their role as biomarkers in psychotic disorders. For example, our current understanding of how antibody status varies over time is poor. For instance, antibody titers may go up and down, possibly in association with psychotic symptom severity. Further longitudinal studies, both of treatment-naïve patients with established psychotic disorders *and* of patients at high clinical risk for the development of such disorders are required to assess the utility of autoantibody measurement in psychotic disorders.

Multiple antibodies have emerged as potential biomarkers of response to atypical treatment strategies (eg, immunotherapies, anti-infective therapies, nutritional or dietary manipulations) in psychotic disorders. Generally, however, randomized controlled trial evidence is lacking or is inconclusive. What is clear is that these antibodies can be an essential part of future study design and stratification of patients into treatment groups. Indeed, failure to stratify patients in this way to date may underlie the limited success of trials of some novel therapies (eg, immunotherapies) in psychotic disorders.

With regard to diagnosis, it is important to consider that psychiatric diagnoses lack pathological specificity. There is an emerging consensus that unlike, say, T1D, NMO or Alzheimer’s disease, “psychosis” denotes a heterogenous group of disorders with likely diverse aetiologies. Some authors have seen psychiatric diagnoses as “manmade abstractions, liable to be discarded or modified,” ^126^ while others have argued that despite the multiplicity of causes, there exists a “final common pathway” that results in the clinical expression of psychosis.^127^ If we accept heterogeneity but also the likely existence of some common pathway, this has important implications for the development of antibody-based biomarkers. It suggests that the “top-down”/single-antigen approach may be unsuccessful in identifying single biomarkers that are diagnostic of a psychotic disorder. Where this approach is more likely to show utility is in the identification of disease subtypes and the consequent implications for treatment stratification. It may be that individual antibodies will be of doubtful diagnostic, prognostic, or predictive significance alone, and that multiple antibodies in combination will guide management. It is possible that this will lead to greater sensitivity and specificity although arguably at the expense of a simple model of the pathophysiology.

Indeed, the possibility of a shared common mechanism despite potentially varied aetiology suggests that a hypothesis-neutral, -omics approach (the “bottom-up” approach) may be an appropriate strategy for predictive biomarker identification going forward.

In parallel to the research outlined here on antibodies as psychosis biomarkers, the emerging immunological perspective on psychotic disorders has suggested that other classes of biomarker, such as cytokines, chemokines, and even metagenomic indices of the microbiome may all have a role in bringing psychosis into the era of personalized medicine. How these measures might relate to antibody serostatus remains unclear although a complex, interactive picture is beginning to emerge from other areas of medicine.^128^

Finally, experience from biomarker identification in other medical disorders has reinforced the value of approaches that combine multiple biomarkers with clinical and demographic data to maximize predictive potential.^7,129,130^ It is likely that where psychotic disorders are concerned, too, the utility of an individual’s antibody profile will be strengthened when used in combination with complementary, non-antibody-based prediction approaches, potentially incorporating neuroimaging, environmental, clinical, genomic, and proteomic data.

## Funding

TAP was supported by a clinical research training fellowship grant from the Wellcome Trust (no 105758/Z/14/Z). JR was supported by a NIHR Academic Clinical Fellowship. RGN has received research funding from the National Institutes of Health, Michael J. Fox Foundation, Osteopathic Heritage Foundation, GlaxoSmithKline, Foundation Venture Capital Group, and the Boye Foundation. He is also a co-founder of Durin Technologies, Inc., serves as its Chief Scientific Officer, and has received consulting fees. He may accrue revenue in the future based on patents submitted by Rowan University wherein he is a co-inventor. There are no marketed products to declare. ASD, JMS, and PM are supported by the NIHR Maudsley Biomedical Research Centre at South London & Maudsley NHS Foundation Trust and the Institute of Psychiatry, Psychology and Neuroscience, King’s College London.

## Conflicts of interest

The authors have declared that there are no conflicts of interest in relation to the subject of this study.
